# Design, Modeling, and Experiment Characterization of a Piezoelectric Inchworm Actuator for Long-Stroke and High-Resolution Positioning

**DOI:** 10.3390/mi17020161

**Published:** 2026-01-27

**Authors:** Xin Li, Zijian Jing, Jin Wang, Fanhui Meng, Xiaoli Yang, Tao Qin, Wei Yan, Bo Qi

**Affiliations:** 1 State Key Laboratory of Optical Field Manipulation Science and Technology, Institute of Optics and Electronics, Chinese Academy of Sciences, Chengdu 610209, China; lixin204@mails.ucas.ac.cn (X.L.);; 2 Key Laboratory of Optical Engineering, Chinese Academy of Sciences, Chengdu 610209, China; 3 University of Chinese Academy of Sciences, Beijing 100049, China; 4 Key Laboratory of High-Performance Manufacturing for Aero Engine, Northwestern Polytechnical University, Ministry of Industry and Information Technology, Xi’an 710072, China; 5 School of Electronics and Information, Northwestern Polytechnical University, Xi’an 710072, China; 6 Engineering Research Center of Advanced Manufacturing Technology for Aero Engine, Northwestern Polytechnical University, Ministry of Education, Xi’an 710072, China

**Keywords:** piezoelectric inchworm actuator, high resolution, self-locking

## Abstract

In this paper, a new type of actuator was presented. The actuator has two modes, stepping and scanning modes. The stepping mode can achieve large stroke through motion accumulation, while the scanning mode can enable high-resolution driving. The mechanical structure and the operating principles of the actuator were described. The structure and the model of the clamping units and driving unit were designed by theoretical modeling and finite element simulation analysis. Finally, a prototype was fabricated, and the experiment results revealed that the actuator achieves a maximum speed of 367 μm/s and a resolution of 0.02 μm.

## 1. Introduction

Precision actuators play a crucial role in fields such as biomedical engineering [1,2,3,4], aerospace, micro-nano manufacturing, and robotics. These fields present significant challenges for actuator design, as it requires precision actuators to achieve both millimeter-scale travel and nanometer-level resolution. Currently, piezoelectric stepping actuators represent one of the best choices to produce a large motion stroke and high resolution.

Piezoelectric stepping drives generally fall into three main categories [5,6,7]: ultrasonic actuators [8,9], stick-slip actuators [10,11], and inchworm actuators [12,13,14]. Among these, the inchworm-type piezoelectric actuators are distinguished by their ability to combine long travel ranges with high-resolution positioning [15,16]. In recent years, numerous scholars have conducted extensive research on inchworm-type piezoelectric actuators. Meng [17] proposed a symmetric clamp feet inchworm actuator with two PZT stacks which can achieve an alternating driving. However, an additional adjustment platform is required during the working progress to regulate the initial gap between the moving platform and the clamping mechanism. Deng [18] developed a compact inchworm piezoelectric actuator including a three-jaw type clamping mechanism, and the actuator achieved a maximum speed of 155.5 μm/s and a maximum thrust of 12.3 N. In the reference paper [19,20,21], the parasitic motion of a rhombic amplification mechanism is utilized for clamping, achieving performance similar to that of a traditional inchworm actuator using only two PZTs. However, since the driving and clamping mechanisms share the PZT, this significantly affects the motion speed. The piezoelectric actuator proposed by Shao employs a hybrid driving mechanism combining the principles of inchworm and stick-slip driving, and the inchworm piezoelectric actuator utilizes an MEMS ridge as the engagement mechanism. It achieves high output force but exhibits relatively low displacement resolution [22,23]. While the aforementioned studies have extensively explored novel designs for both driving and clamping mechanisms, they have largely overlooked the friction phenomenon within the actuators. Since the working principle of inchworm actuators relies fundamentally on friction, the modeling of friction constitutes the core of their dynamic modeling.

This paper presents an inchworm piezoelectric actuator capable of operating in either scanning or stepping mode. In stepping mode, it enables large stroke through motion accumulation. In scanning mode, the high resolution of the actuator can be utilized to achieve high-resolution actuation of the mechanism. The switching between the two modes is accomplished by altering the electrical signals of PZTs, and it can be seamlessly and instantly switched at any time. The friction between the driving unit and the clamping unit was calculated, and a dynamic model of the driving unit was developed.

The remainder of this paper is organized as follows. The detailed structural design and working principle of the actuator will be presented in Section 2. Static modeling and finite element analysis will be carried out in Section 3 to evaluate its theoretical performance. Experimental tests on motion characteristics will be performed in Section 4 to validate the prototype and analyze the results, and Section 5 will conclude the paper.

## 2. Structural Design and Operating Principle

### 2.1. Mechanical Structural Design of the Actuator

**Figure 1:**
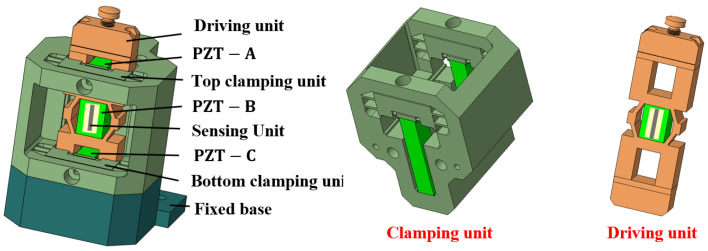
Structure of the proposed actuator.

The proposed actuator consists of two clamping units, a driving unit, and a fixed base, as shown in Figure 1. The clamping units are designed with a double-layer flexible hinge structure, with the inner layer flexible hinge used for mounting the PZT-A and PZT-C. The outer layer flexible hinge is used for mounting and clamping–releasing the driving unit. The inner flexible hinge has a clearance fit with the PZT and is preloaded using screws. The clamping unit has an interference fit with the drive unit. During installation, screw rotation is employed to generate targeted flexure hinge deformation, which creates sufficient clearance between the clamping units and driving unit interfaces for assembly purposes.

Upon application of a high-level voltage signal to excite the PZT-A or PZT-C at the clamping unit, the outer layer of the flexible hinge at the clamping unit deforms under force and moves away from the driving unit. The clamping preload is determined by the interference fit between the clamping units and the driving unit, with greater interference resulting in higher preload forces. To ensure sufficient actuation force and displacement for disengagement, the clamping unit incorporates piezoelectric stacks with high thrust capacity and output displacement. In the de-energized state, the clamping unit maintains secure contact with the driving unit through interference fit, where the substantial preload force ensures reliable self-locking capability. The two clamping units feature a monolithic design, with the top and bottom clamping units rigidly connected via the frame and manufactured as an integrated component. This design and fabrication approach significantly reduces assembly errors between the clamping units. The driving unit incorporates hollow rectangular structures at both ends, within which the PZTs of the two clamping units are vertically positioned. A rhombic flexure structure at the center provides preload force, firmly securing PZT-C at the driving unit location. Bonded to the side wall of the PZT, the strain gauge functions as the feedback sensor.

### 2.2. Operating Principle of the Actuator

The proposed actuator has two operating modes: stepping and scanning.

**Figure 2:**
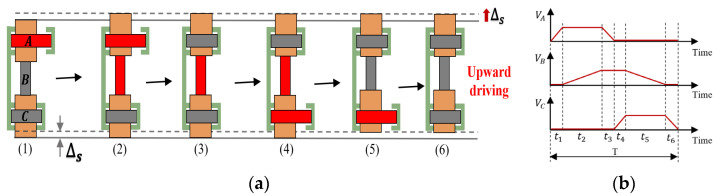
Stepping mode of the proposed actuator: (**a**) the working principles of the actuator in stepping mode; (**b**) the sequential waveform of the input voltage signal.

Figure 2a illustrates the working principles of the actuator in stepping mode, respectively, while Figure 2b illustrates the sequential waveform of the input voltage signal. In the initial state, the clamping unit tightly presses against the driving unit to achieve self-locking. A working cycle consists of the following six steps:

(1) PZT-A energized and extended, the top clamping unit leaves the driving unit.
(2) PZT-B energized and extended, the driving unit produces an upward displacement of magnitude ∆s.
(3) PZT-A deenergized, the actuator contracts back to the length it had before the high voltage was applied under its own elastic force.
(4) PZT-C energized and extended, the bottom clamping unit leaves the driving unit.
(5) PZT-B deenergized, the actuator contracts back to the length it had before the high voltage was applied.
(6) PZT-C deenergized, the actuator contracts back to the length it had before the high voltage was applied. The actuator completes a single feed motion, outputting displacement ∆s.

By cycling continuously through these steps, the actuator can accumulate single-step displacement to achieve large-stroke motion. The downward driving principle is analogous to that of upward motion. By reversing the sequence of the input voltage signals, rapid switching between upward and downward can be achieved.

**Figure 3:**
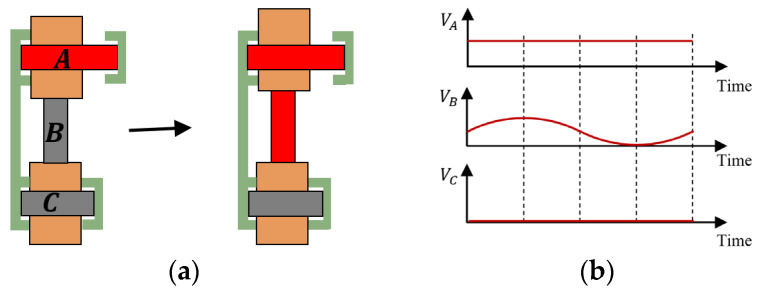
Scanning mode of the proposed actuator: (**a**) the working principles of the actuator in scanning mode; (**b**) the sequential waveform of the input voltage signal.

The Figure 3 presents the working principle of the actuator when the actuator work in scanning mode.

(1) PZT-A extended, the top clamping unit leaves the driving unit.
(2) PZT-B generates controlled micro-displacement when driven by a voltage-modulated sinusoidal waveform, enabling precise motion regulation through electrical input adjustment.

Each step in stepping mode corresponds to a full-stroke movement in scanning mode.

## 3. Structural Modeling and Analysis

The actuator’s mechanical output depends on how far the PZT stack can expand and on how effectively the clamping and driving stages operate. A combination of finite element simulation (FEM) and theoretical analysis is used to study the stiffness and deformation of the flexible hinge of the clamping unit, as well as the relationship between the output displacement of the driving unit and the excitation voltage applied to the PZT stack, offering a theoretical basis for designing the actuator. Additionally, a dynamic model was established to characterize the system’s performance.

### 3.1. FEM of the PZT Stack

The PZT stacks are selected based on distinct performance requirements corresponding to their functional positions within the actuator: (a) PZT stacks for clamping units, which require maximum thrust and output displacement to ensure the clamping units can fully release the driving unit; and (b) PZT stacks for drives, which require high thrust and resolution while minimizing volume to reduce the overall actuator size. Based on the above two requirements, PZT stacks with dimensions of 7 mm × 7 mm × 36 mm and 10 mm × 10 mm × 13.5 mm are used for the clamping units and driving unit, (P-887.91, P-888.31, from Harbin Core Tomorrow Science & Technology Co., Ltd., Harbin, China), as shown in Table 1.

**Figure 4:**
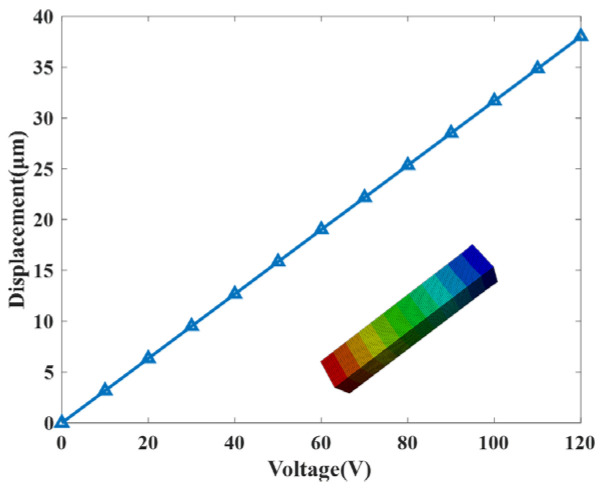
Deformation of PZT stack versus excitation voltage.

A PZT stack is composed of multiple piezoelectric ceramic layers alternating with electrode layers, configured in a mechanical series yet electrically parallel arrangement. This design allows the stack to generate substantial displacement under relatively low driving voltages. Using a PZT stack model measuring 7 mm × 7 mm × 36 mm as an example, its displacement response under varying excitation voltages was investigated via finite element analysis. The model was discretized using hexahedral elements with a size of 0.3 mm. One end of the stack was fixed, while the other remained free. Voltage was applied along the polarization direction, and the resulting axial deformation at the free end was recorded. As shown in Figure 4, the axial deformation exhibits a nearly linear relationship with the excitation voltage. At 120 V, the simulated displacement reaches 38.02 μm.

### 3.2. Modeling and Analysis of Clamping Units

**Figure 5:**
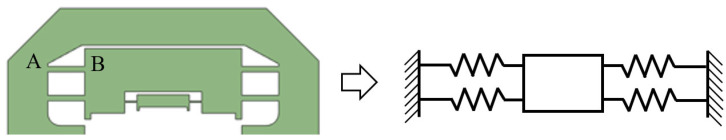
Simplified schematic diagram of the clamping unit.

The clamping units must satisfy two critical mechanical criteria: (a) structural stiffness—the unit must exhibit sufficient rigidity in the clamping direction to generate the required preload force between the clamping units and driving unit; and (b) stress resistance—to prevent plastic deformation or fracture during operation, the maximum bending stress must not exceed the conditional yield strength of the material. Following the pseudo-rigid-body modeling approach, deformation is assumed to be confined to the flexure hinges and beams, while the links and fixed frame are treated as rigid bodies. The flexible portion of the clamping unit can be regarded as a symmetric parallel spring model, with its structural schematic shown in Figure 5.

**Figure 6:**
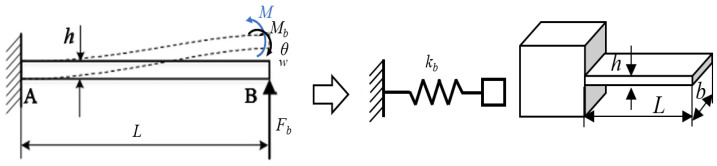
Simplification of flexible beam.

This section uses the flexible arm AB as an example to establish the simplified model shown in Figure 6. In this model, when the PZT-A or PZT-C at the clamping unit position is activated, under elastic deformation, AB is idealized as an elastic beam having an axial stiffness *k_b_*, where *F_b_* and M denote the vertical force and constraint moment at point B, respectively, *θ* and w are the angle of rotation and deflection at point B, respectively, and the moments *L*, *b*, and *h* represent the length, width, and thickness of the flexible arm AB, respectively.

Under normal circumstances, the free end of a cantilever beam will have an angle $\theta (L)=F_{b}L^{2}/(2EI)$ when subjected to a concentrated force, but in this model, the free end (point B) maintains zero angular displacement (*θ_B_* = 0). This constraint is enforced through an applied reaction moment *M* to offset the rotation caused by force *F_b_*.

Based on the principle of superposition, the angle of rotation and deflection at point B:

(1) $$\left\{\begin{array}{l} \theta_{F_{b}}(L)=\frac{F_{b}L^{2}}{2EI}\\ w_{F_{b}}(L)=\frac{F_{b}L^{3}}{3EI} \end{array}\right.$$

Angular displacement and deflection caused by torque *M*:

(2) $$\left\{\begin{array}{l} \theta_{M}(L)=\frac{ML}{EI}\\ w_{M}(L)=\frac{ML^{2}}{2EI} \end{array}\right.$$

In Equations (1) and (2), *I* is the second moment of area in beam analysis.

The total rotation angle and deflection of the free end (point B) can be expressed as

(3) $$\left\{\begin{array}{l} \theta (L)=\theta_{F_{b}}(L)+\theta_{M}(L)=\frac{F_{b}L^{2}}{2EI}+\frac{ML}{EI}=0\\ w(L)=w_{F_{b}}(L)+w_{M}(L)=\frac{F_{b}L^{3}}{3EI}+\frac{ML^{2}}{2EI} \end{array}\right.$$

From Equation (3), we can obtain the constraint torque *M*:

(4) $$M=-\frac{FL}{2}$$

where the negative sign denotes that the direction of *M* is opposite to the *M_b_* direction caused by *F_b_*.

Free end (point B) deflection:

(5) $$w(L)=\frac{F_{b}L^{3}}{3EI}+\frac{(-FL/2)L^{2}}{2EI}=\frac{F_{b}L^{3}}{12EI}$$

Displacement stiffness of flexible arm AB:

(6) $$k_{b}=\frac{F_{b}}{w(L)}=\frac{F_{b}}{F_{b}L^{3}/12EI}=\frac{12EI}{L^{3}}$$

(7) $$I=\frac{bh^{3}}{12}$$

(8) $$k_{b}=\frac{Ebh^{3}}{L^{3}}$$

Bending moment equation *M*(*x*) caused by the combined action of force *F_b_* and moment *M*:

(9) $$M(x)=F_{b}(L-x)+M=F_{b}\left(\frac{L}{2}-x\right)$$

where the fixed end (point A) is $(x=0):M(0)=F_{b}L/2$, the free end (point B) is $(x=L):M(L)=-F_{b}L/2$, and the midpoint is (x=L/2):M(L/2)=0

Bending stress σ(x, y):

(10) $$\sigma (x,y)=\frac{M(x)\cdot y}{I},y\in [-h/2,h/2]$$

The maximum stress occurs at the fixed end (point A) and the outermost fiber (y=±h/2):

(11) $$\sigma_{\max}=\frac{\left(F_{b}L/2)\cdot (h/2\right)}{I}=\frac{F_{b}Lh}{4I}=\frac{3F_{b}L}{bh^{2}}$$

**Figure 7:**
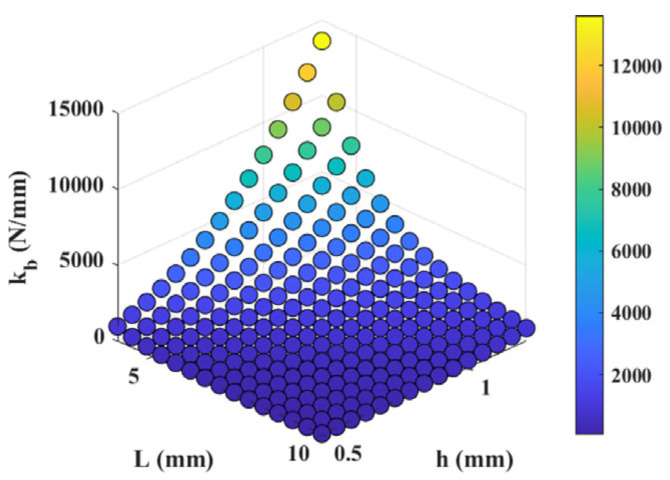
The influence of the external flexible hinge size parameters of the clamping unit on stiffness.

Equations (8) and (11) indicate that the parameters affecting the stiffness and bending stress of the clamping unit are *L*, *b*, and *h*. In the static analysis, the entire structure except for the PZT is set to be 7075 aluminum alloy. Considering the need for dimensional compatibility with the piezoelectric stack, the parameter b is set to 7 mm. Figure 7 shows the influence of the outer layer flexible hinge size parameters on the stiffness. The stiffness of the clamping unit decreases as the length of the outer layer flexible hinge increases and increases as the thickness increases.

The magnitude of the clamping force between the clamping unit and the driving unit is related to factors such as the interference amount between the two units and the stiffness of the outer flexible hinge of the clamping unit. The expected designed clamping force is greater than 120 N, and the interference amount is approximately 0.03 mm. Then, the stiffness of the outer flexible hinge needs to be greater than 4000 N/mm, and the stiffness of a single flexible arm needs to be greater than 1000 N/mm. Among the numerous feasible data, we choose L = 6 mm and h = 0.9 mm to enter the design stage.

The final design of the clamping unit resulted from extensive static analysis. Consequently, the dimensions are finalized as *L* = 6 mm, *b* = 7 mm, and *h* = 0.9 mm.

**Figure 8:**
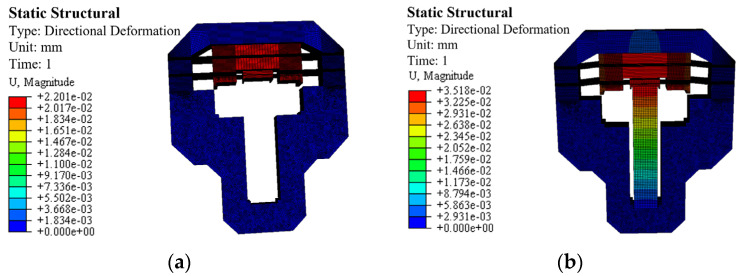
Static analysis results of the clamping unit: (**a**) deformation result when applying a pressure of 100 N on the outer flexible hinge of the clamping unit, (**b**) deformation result when applying a voltage of 120 V to the piezoelectric stack.

Finally, a finite element model is established based on the final parameters. All contact interactions in the finite element model are defined as adhesive. Except for PZT, the entire structure is set as 7075 aluminum alloy, with a density of 2810 kg/m^3^ and Young’s modulus of 72 GPa, and a Poisson’s ratio of 0.33. The density of PZT is 7500 kg/m^3^, with other parameters as shown in Table 1. The finite element model is meshed using the hexahedral dominant method. Figure 8a shows the deformation of the outer flexure hinge under a 100 N preload with fixed external boundaries. It can be found that the output displacement of the outer flexure hinge is 22.01 µm, thus, the equivalent stiffness of 4.54 N/µm in the clamping direction, indicating high structural rigidity. The deformation of the clamping unit is shown in Figure 8b. When a voltage of 120 V is applied to the piezoelectric stack, the output displacement of the outer flexible hinge is 35.18 µm. Therefore, when the piezoelectric stack is powered off, the maximum clamping force between the clamping unit and the driving unit theoretically is 159.84 N. When the friction coefficient is 0.15, the clamping unit can provide a static clamping friction force of 24.98 N, enabling a significant output force to be achieved.

**Figure 9:**
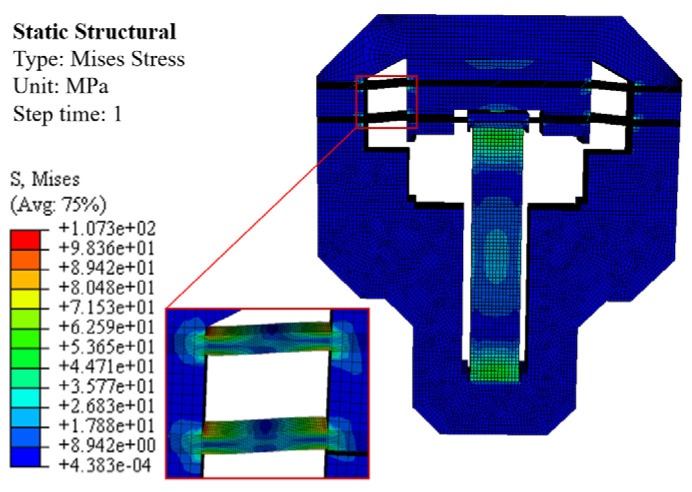
Simulation stress diagram of the clamping unit.

Under a voltage of 120 V, the static simulation stress of the proposed actuator is shown in Figure 9. The maximum stress occurs in the four flexible arm sections, with a maximum stress value of 107.3 MPa, which is far below the material’s allowable stress value (455 MPa). The calculated result is compared with the theoretical value of 107.78 MPa, with a deviation of 0.45%.

### 3.3. Modeling and Analysis of Driving Unit

The output characteristics of the actuator in scanning mode are predominantly governed by the design of its driving unit. Therefore, to understand this influence, the relationship between the unit’s output displacement and the PZT stack excitation voltage is analyzed in this section.

**Figure 10:**
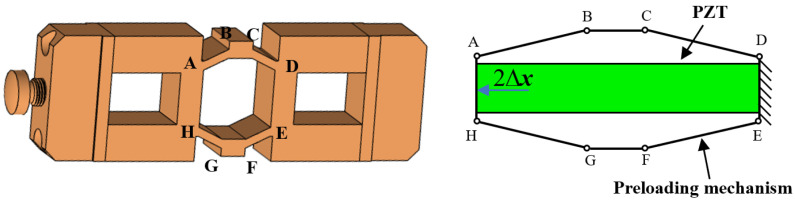
Simplified schematic diagram of the PZT and the preloading mechanism.

The driving unit is modeled as a piezoelectric stack (P-888.31) combined with a preload mechanism for upward actuation. Taking the elongation of the PZT as an example, Figure 10 shows a simplified diagram of the PZT stack and preload mechanism.

**Figure 11:**
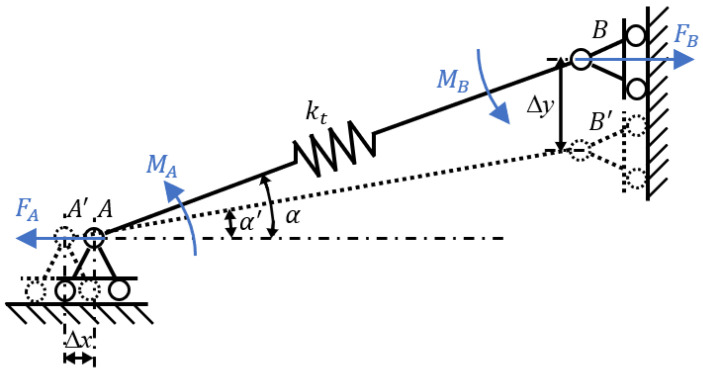
Schematic of the force and moments applied on rod AB.

Considering that the preload mechanism has a symmetrical structure, this section only analyzes the force conditions of the AB rod. When the preload mechanism is actuated, rod AB deforms elastically, so it is treated as a beam with axial stiffness *k_t_*. The corresponding forces and moments acting on the rod are illustrated in Figure 11.

In the model, points A and B are defined as torsional spring hinges that share the same rotational stiffness *k_r_*. In the initial configuration, rod AB is inclined at an angle α to the horizontal. Upon application of a driving voltage to the PZT stack, point A displaces leftward by ∆x under the thrust force *F_A_*, while point B moves downward by ∆y under tension *F_B_*. The resulting elastic deformation along the length of AB is denoted as $\Delta L_{AB}$. The following relationship can be established:

(12) $$\left\{\begin{array}{l} F_{A}=F_{B}\\ M_{A}=M_{B}\\ 2M_{A}=2k_{r}\left(\alpha -\alpha^{\prime }\right)\\ k_{t}\Delta L_{\mathrm{AB}}=F_{B}\cos\alpha \end{array}\right.$$

where *M_A_* and *M_B_* denote the bending moments at torsional hinges A and B, respectively, $\alpha^{\prime }$ represents the final inclination angle of rod AB relative to the horizontal, and L is the undeformed length of the rod.

Applying the principle of virtual work yields the following relation [22,24]:

(13) $$F_{A}\Delta x=2M_{A}(\alpha -\alpha^{\prime })+F_{t}\Delta L_{\mathrm{AB}}$$

where *F_t_* denotes the elastic force developed in rod AB.

(14) $$F_{t}=k_{t}\Delta L_{\mathrm{AB}}=F_{A}\cos\alpha$$

By combining Equations (12) and (14), the displacement at point A is obtained as

(15) $$\Delta x=L_{\mathrm{AB}}\sin\alpha \Delta \alpha +\cos\alpha \Delta L_{\mathrm{AB}}$$

Subsequently, the free axial deformation of the PZT stack is determined from the following expression:

(16) $$\delta_{0}=nd_{33}U$$

where $d_{33}$ the piezoelectric strain constant, *n* denotes the number of layers in the PZT stack, and *U* represents the applied voltage.

During operation, the axial deformation of the bonded PZT within the preload assembly is significantly influenced by the mechanical characteristics of the preload mechanism. The axial stiffness of this preload mechanism can be analytically derived as follows:

(17) $$k_{\mathrm{pre}}=\frac{F_{A}}{2\Delta x}=\frac{k_{r}k_{t}}{k_{t}L_{\mathrm{AB}}^{2}\sin^{2}\alpha +2k_{r}\cos^{2}\alpha }$$

where $k_{pre}$ is the stiffness of the preload mechanism.

When PZT is stacked under excitation voltage U, the single-step displacement of the entire driving unit is determined using the following relation:

(18) $$\Delta x=\frac{k_{\mathrm{PZT}}}{k_{\mathrm{PZT}}+k_{\mathrm{Pre}}}\delta_{0}$$

where $k_{PZT}$ is the stiffness of the PZT stack.

**Figure 12:**
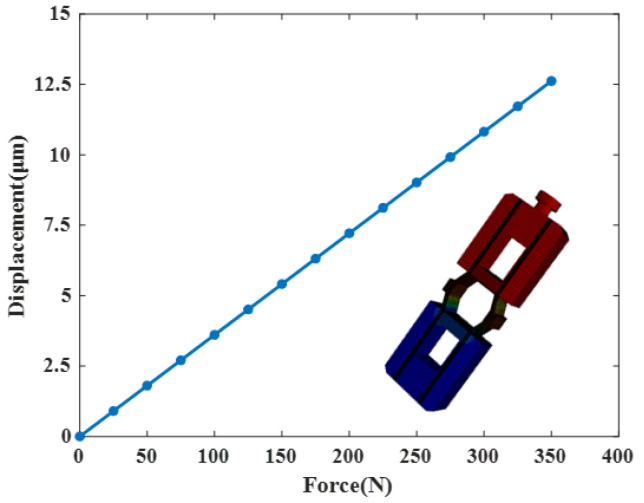
The force–displacement characteristic of the amplifying mechanism.

The stiffness of the PZT is $k_{PZT}=267N/\mu m$. Using finite element analysis software, the axial stiffness of the preload mechanism is determined, as shown in Figure 12. The material is 7075 aluminum alloy, with a density of 2810 kg/m^3^, a Young’s modulus of 72 GPa, and a Poisson’s ratio of 0.33. The key geometric parameters of the preload mechanism in the driving unit are *L*_AB_ is 5 mm and α is 25.69°.

### 3.4. Friction Calculation and Dynamic Model of the Actuator

The friction force between the clamping unit and the driving unit is a key factor affecting the performance of the actuator. The friction force is determined by the clamping force and the coefficient of friction. The clamping force is generated by PZT-A and PZT-C:

(19) $$\left\{\begin{array}{l} N_{t}=F_{clamp,t}=F_{clamp,0}-k_{PZT-A}\;\cdot V_{A}\\ N_{b}=F_{clamp,b}=F_{clamp,0}-k_{PZT-C}\;\cdot V_{C} \end{array}\right.$$

where *N_t_* and *N_b_* are the normal forces exerted by the clamping units, $F_{clamp,t}$ and $F_{clamp,b}$ are the clamping forces generated by PZT-A and PZT-C, $F_{clamp,0}$ indicates the initial preload force of the clamping unit when neither PZT-A nor PZT-C is energized, and $k_{PZT-A}$ and $k_{PZT-B}$ are the proportional coefficients for PZT-A and PZT-C. Since PZT-A and PZT-C share the same model, $k_{PZT-A}=k_{PZT-B}$.

Taking two typical nodes in one stepping cycle (“*the bottom clamping unit is clamped, the top clamping unit is released, and PZT-B retains its original length*” and “*the bottom clamping unit is clamped, the top clamping unit is released, and PZT-B is energized to generate a driving force* $F_{driving}$ ”) as examples, we will analyze the friction as follows:

Node 1: *The bottom clamping unit is clamped, the top clamping unit is released, and PZT-B retains its original length*. At this point, $V_{C}=0,N_{b}=F_{clamp,0},N_{t}=0$

(20) $$\left\{\begin{array}{l} F_{f,b}=\mu_{s}N_{b}\\ F_{f,t}=0 \end{array}\right.$$

where $\mu_{s}$ is the static friction coefficient, $F_{f,b}$ is the friction between the bottom clamping unit and the driving unit, and $F_{f,t}$ is the friction between the top clamping unit and the driving unit.

Node 2: *The bottom clamping unit is clamped, the top clamping unit is released, and PZT-B is energized to generate a driving force* $F_{driving}$. At this point, $V_{C}=0,N_{b}=F_{clamp,0},N_{t}=0$.

(21) $$m_{d}\;{\ddot{x}}_{d}\;=F_{driving}\;+F_{f,t\;}+F_{f,b}$$

where $m_{d}$ is the mass of the driving unit and $x_{d}$ is the displacement of the driving unit relative to the fixed base. $F_{f,t}=0$, $F_{f,b}$ is employed to prevent sliding between the driving unit and the bottom clamping unit. When the driving unit is in a quasi-static state, i.e., ${\ddot{x}}_{d}\approx 0,F_{driving}=-F_{f,b}$. If $\left|F_{driving}\right|<\mu_{s}N_{b}$, it means that the slipping phenomenon between the driving unit and the bottom clamping unit does not appear. The output displacement of the driving unit is given by

(22) $$x_{d}=\Delta x=\frac{k_{\mathrm{PZT}}}{k_{\mathrm{PZT}}+k_{\mathrm{Pre}}}\delta_{0}$$

If $\left|F_{driving}\right|>\mu_{s}N_{b}$, the slipping phenomenon occurs between the driving unit and the bottom clamping unit. The formula for calculating the sliding displacement can be written as follows:

(23) $$F_{driving}-\mu_{s}N_{b}=m_{d}{\ddot{x}}_{s}$$

where $x_{s}$ is the sliding displacement.

From the single-step displacement and the driving voltage frequency, the actuator speed is obtained:

(24) $$v=x_{d}f_{V}$$

where $f_{V}$ is the driving voltage frequency.

The above analysis simplifies the entire model into a simple ideal model, considering friction as only two states—“static friction” and “sliding friction”—and the sliding friction coefficient $\mu_{s}$ as a constant. The complex microscopic transition process from rest to motion is ignored. On this basis, in order to more accurately analyze the friction force between the clamping unit and the driving unit and the dynamic model of the driving unit, next, we will use the LuGre model to analyze the friction force.

The LuGre model regards the contact surface as an elastic deformation of bristles.

Taking into account the relative motion between the two contact surfaces, the relative velocity is $v_{rel}$. The core of the LuGre model is an internal state variable *z* (the average deformation of the bristle), and its dynamic equation is as follows:

(25) $$\frac{dz}{dt}=v_{rel}-\frac{\left|v_{rel}\right|}{g\left(v_{rel}\right)}z$$

where the function $g(v_{rel})$ describes the Stribeck effect, which usually takes the form of

(26) $$g\left(v_{rel}\right)=\frac{1}{\sigma_{0}}\left[\mu_{k}+\left(\mu_{s}-\mu_{k}\right)e^{-{\left|v_{rel}/v_{s}\right|}^{\gamma }}\right]N$$

(27) $$g(v_{rel})=\frac{1}{\sigma_{0}}F_{c}+(F_{s}-F_{c})e^{-{\left(v_{rel}/v_{s}\right)}^{2}}$$

where $F_{c}$ and $F_{s}$, respectively, represent the static friction force and the sliding friction force and $v_{s}$ is the Stribeck speed: $F_{c}=\mu_{k}N$, $F_{s}=\mu_{s}N$.

Friction force $F_{f}$:

(28) $$F_{f}=\sigma_{0}z+\sigma_{1}\frac{dz}{dt}+\sigma_{2}v_{rel}$$

where $\sigma_{0}$ is the stiffness of the bristle, $\sigma_{1}$ is the microscopic damping coefficient, and $\sigma_{2}$ is the viscous friction coefficient.

The complete equation of the LuGre model is as follows:

(29) $$\dot{z}=v_{rel}-\frac{\left|v_{rel}\right|}{g\left(v_{rel}\right)}z=v_{rel}-\sigma_{0}\frac{\left|v_{rel}\right|}{N\left[\mu_{k}+\left(\mu_{s}-\mu_{k}\right)e^{-{\left(v_{rel}/v_{s}\right)}^{2}}\right]}z$$

(30) $$F_{f}=\sigma_{0}z+\sigma_{1}\dot{z}+\sigma_{2}v_{rel}$$

Top contact surface model:

(31) $$v_{rel,t}=v_{d}-v_{t}$$

(32) $$\frac{dz_{t}}{dt}=v_{rel,t}-\sigma_{0}^{t}\frac{\left|v_{rel,t}\right|}{g_{t}\left(v_{rel,t}\right)}z_{t}$$

(33) $$g_{t}(v_{rel,t})=\frac{N_{t}(t)}{\sigma_{0}^{t}}\left[\mu_{k}^{t}+(\mu_{s}^{t}-\mu_{k}^{t})e^{-{\left(|v_{rel,t}|/v_{s}^{t}\right)}^{2}}\right]$$

(34) $$F_{f,t}=\sigma_{0}^{t}z_{t}+\sigma_{1}^{t}\frac{dz_{t}}{dt}+\sigma_{2}^{t}v_{rel,t}$$

Bottom contact surface model:

(35) $$v_{rel,b}=v_{d}-v_{b}$$

(36) $$\frac{dz_{b}}{dt}=v_{rel,b}-\sigma_{0}^{b}\frac{\left|v_{rel,b}\right|}{g_{b}\left(v_{rel,b}\right)}z_{b}$$

(37) $$g_{b}(v_{rel,b})=\frac{N_{b}(t)}{\sigma_{0}^{b}}\left[\mu_{k}^{b}+(\mu_{s}^{b}-\mu_{k}^{b})e^{-{\left(|v_{rel,b}|/v_{s}^{b}\right)}^{2}}\right]$$

(38) $$F_{f,b}=\sigma_{0}^{b}z_{b}+\sigma_{1}^{b}\frac{dz_{b}}{dt}+\sigma_{2}^{b}v_{rel,b}$$

Embed the LuGre model into the previously established dynamic system, and the state vector is expanded to 8 dimensions:

(39) $$X={\left[x_{t},v_{t},x_{d},v_{d},x_{b},v_{b},z_{t},z_{b}\right]}^{T}$$

where $x_{t}$ and $x_{b}$ are the displacements of the top and bottom clamping units, $v_{t}$ and $v_{b}$ are the velocities of the top and bottom clamping units, and $z_{t}$ and $z_{b}$ are the internal state variables of the top and bottom clamping units.

Top clamping unit dynamics:

(40) $$m_{t}{\dot{v}}_{t}=k_{a}(x_{d}-x_{t})+c_{a}(v_{d}-v_{t})-F_{f,t}$$

where $m_{t}$ is the mass of the top clamping unit and $k_{a}$ and $c_{a}$ are the stiffness coefficient and damping coefficient from PZT-A.

Driving unit dynamics:

(41) $$m_{d}{\dot{v}}_{d}=F_{b}(t)-k_{a}(x_{d}-x_{t})-c_{a}(v_{d}-v_{t})+k_{c}(x_{b}-x_{d})+c_{c}(v_{b}-v_{d})+F_{f,t}+F_{f,b}$$

where $k_{c}$ and $c_{c}$ are the stiffness coefficient and damping coefficient from PZT-C.

Bottom clamping unit dynamics:

(42) $$m_{b}{\dot{v}}_{b}=-k_{c}(x_{d}-x_{b})-c_{c}(v_{d}-v_{b})-F_{f,b}$$

where $m_{b}$ is the mass of the bottom clamping unit.

Internal state equation:

(43) $$\begin{array}{l} {\dot{z}}_{t}=v_{rel,t}-\sigma_{0}^{t}\frac{\left|v_{rel,t}\right|}{g_{t}(v_{rel,t})}z_{t}\\ {\dot{z}}_{b}=v_{rel,b}-\sigma_{0}^{b}\frac{\left|v_{rel,b}\right|}{g_{b}(v_{rel,b})}z_{b} \end{array}$$

Pre-slip stiffness:

(44) $$k_{\mathrm{pre}}=\sigma_{0}N\;\;\;\;\left(\;v_{\mathrm{rel}}\approx 0\right)$$

The LuGre model exhibits a memory effect. The friction force not only depends on the current speed, but also on the historical movement.

(45) $$F_{f}(t)=\sigma_{0}\int_{0}^{t}\left[v_{rel}(\tau )-\frac{\left|v_{rel}(\tau )\right|}{g(v_{rel}(\tau ))}z(\tau )\right]d\tau$$

Take the case of the drive unit extending (with the bottom clamped and the top released) as an example, and conduct a dynamic analysis:

Control condition: *N_b_* is large, $N_{t}\approx 0,\;\;\;\;F_{b}>0$

For the top contact surface: $N_{t}\approx 0,\;\;\;\;g_{t}\approx 0$

(46) $$\begin{array}{l} {\dot{z}}_{t}\approx v_{rel,t}-\infty \cdot z_{t}\;\;\;\;\Rightarrow \;\;\;\;z_{t}\to 0\\ F_{f,t}\approx \sigma_{2}^{t}v_{rel,t}\;\;\;\; \end{array}$$

For the bottom contact surface: $N_{b}$ is large.

Initially, $v_{rel,b}=0$, and the state $z_{b}$ gradually accumulates:

(47) $${\dot{z}}_{b}=-\sigma_{0}^{b}\frac{\left|v_{\mathrm{rel},b}\right|}{g_{b}(0)}z_{b}=0$$

When $F_{b}$ increases and $z_{b}$ also increases, but $v_{rel,b}$ remains 0, until

(48) $$\sigma_{0}^{b}z_{b}=F_{s}^{b}=\mu_{s}^{b}N_{b}$$

Once this limit is exceeded, macroscopic sliding begins, and the friction force drops to sliding friction.

No-slip condition:

(49) $$\max(F_{b})<\mu_{s}^{b}N_{b}$$

## 4. Characteristic Experiments of the Actuator

**Figure 13:**
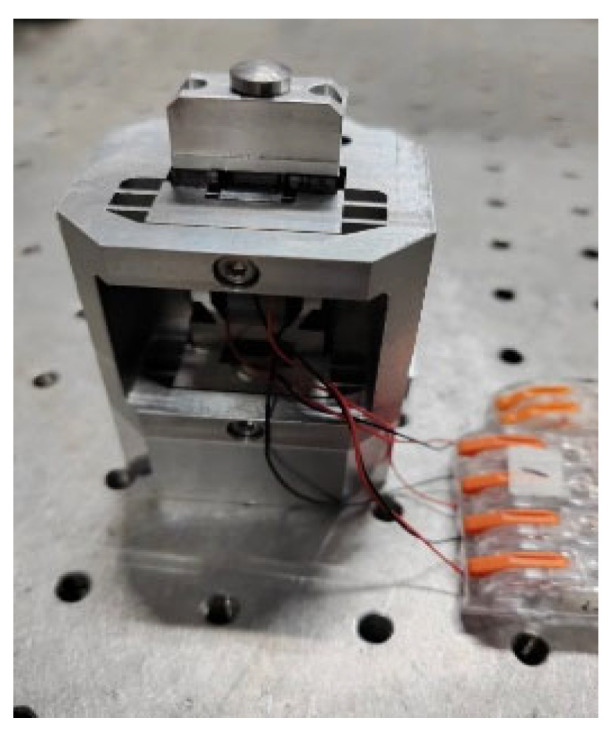
Prototype of the proposed actuator.

**Figure 14:**
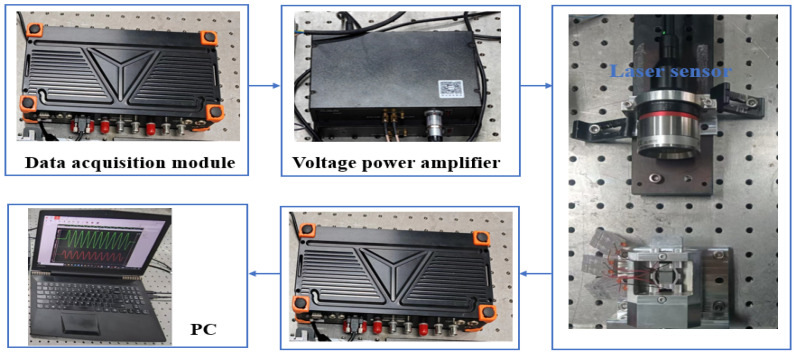
The experimental set-up for the prototype.

Figure 13 shows the prototype of the proposed driver. Figure 14 depicts the experimental set-up used to evaluate the driver’s performance. The Dewesoft data acquisition equipment (Type: SIRIUSi-8xSTG-8xAo) is used to generate the excitation signal shown in Figure 2b and Figure 3b, which is further amplified by the power amplifier (Type: E70.C3K (Harbin Core Tomorrow Science & Technology Co., Ltd., China)) to produce the drive voltage for the actuator. The response displacement of the actuator is measured using a laser displacement sensor equipment (Type: KEYENCE CL-P070), recorded by the data acquisition module, and processed using a PC.

**Figure 15:**
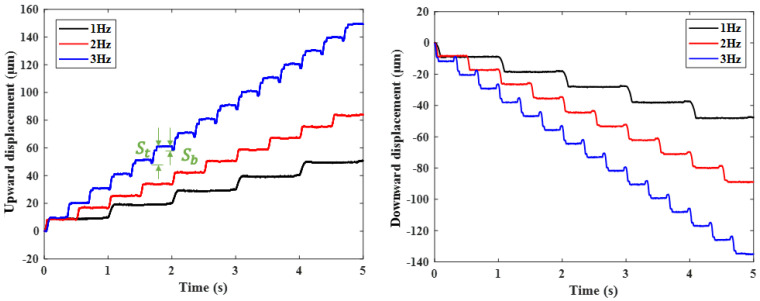
Bidirectional motion curves under different voltages.

Figure 15 shows the step response characteristics of bidirectional drive at different operating frequencies when the drive voltage is 120 V. The drive displacement increases gradually with the drive frequency. The effective step size of the drive is approximately (*S_t_ − S_b_*). However, during bidirectional driving, the step distances for upward and downward motion are inconsistent, primarily due to inconsistent contact states between the clamping units and the driving unit caused by manufacturing and assembly errors. Additionally, the driver exhibits a backward movement phenomenon throughout the stepping motion process, with the severity increasing as the frequency rises.

**Figure 16:**
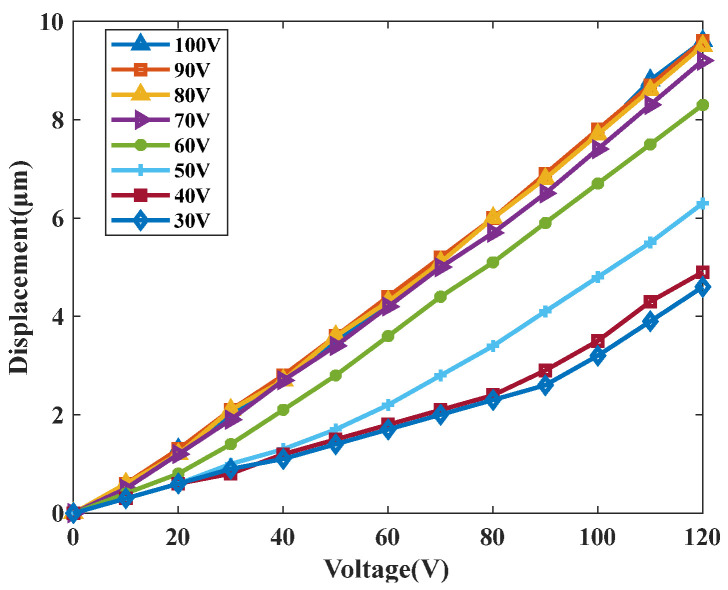
The relationship between the single-step increment of the driving unit and the excitation voltage.

The single-step increment of the drive unit was characterized as a function of the excitation voltage, with the results shown in Figure 16. Additionally, a series of voltages ranging from 30 V to 100 V were applied to the PZT in the clamping unit to investigate their effect on the single-step increment. The experimental results indicate that as the excitation voltage of the drive unit increases, the single-step pitch of the drive unit increases when the excitation voltage of the clamping unit is between 30 V and 90 V. When the excitation voltage of the clamping unit exceeds 90 V, it no longer affects the single-step pitch of the driving unit. When the excitation voltage of the driving unit is 120 V and the excitation voltage of the clamping unit is 90 V, the single-step pitch of the driving unit is maximized at 9.6 μm.

**Figure 17:**
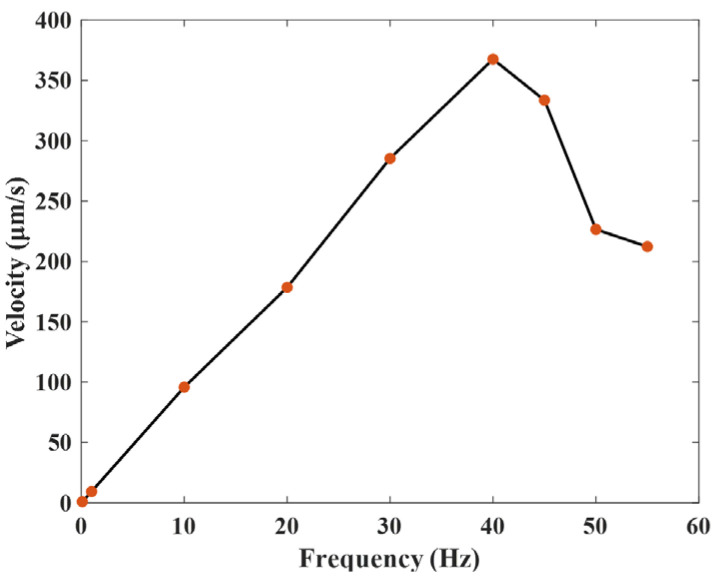
Output speed under different frequencies.

Figure 17 presents the measured maximum output speed of the actuator as a function of frequency. The findings reveal that when the frequency varies between 1 and 40 Hz, the speed increases approximately linearly with frequency. Between 40 and 55 Hz, the speed decreases. At a frequency of 40 Hz, the output speed is at its maximum, reaching 367 μm/s.

**Figure 18:**
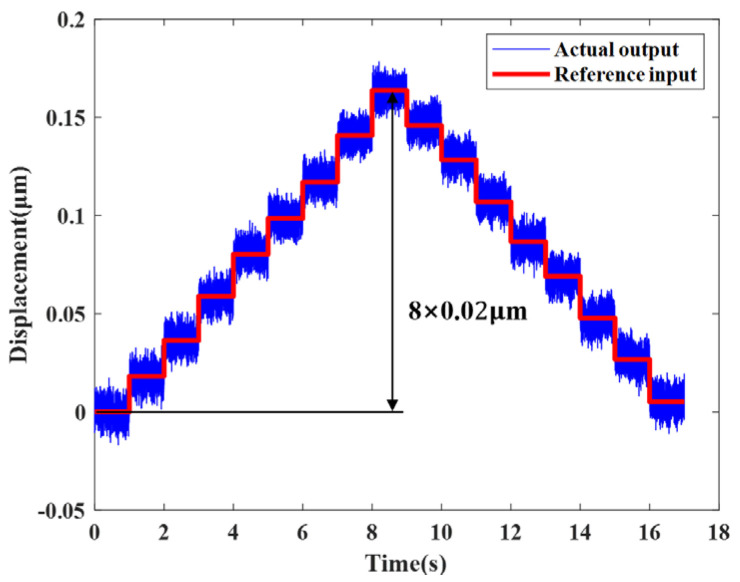
Motion resolution of the actuator.

The resolution of the prototype was experimentally characterized, as illustrated in Figure 18. A stepwise excitation with 0.5 V increments was applied to PZT-B, and the resulting strain from the sensing unit was converted into displacement via a data acquisition module. The measured resolution of the actuator is approximately 0.02 μm.

**Figure 19:**
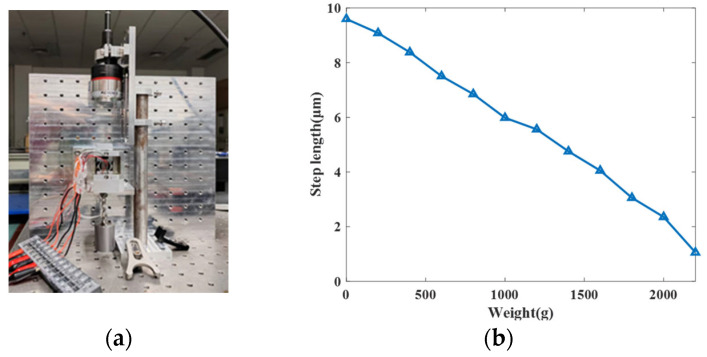
Load capacity experimental (**a**) set-up, (**b**) the result of the proposed actuator.

The step size of the piezoelectric actuator under different loads was tested to study its output force characteristics. The measurement device is shown in Figure 19a. A signal with a frequency of 1 Hz and a voltage of 120 V was applied to the driving unit. The measurement results are shown in Figure 19b. It can be seen that as the load increases, the step size gradually decreases. When the load reaches 2200 g, the proposed driver can still drive at a step size of 1.054 μm, which means that the carrying capacity of the piezoelectric actuator is estimated to be greater than 22 N.

Table 2 compares the proposed actuator and other works presented in recent years. The comparison demonstrates its high resolution, achieved without requiring additional guiding structures.

## 5. Conclusions

This paper introduces an inchworm piezoelectric actuator capable of operating in two distinct modes: scanning and stepping modes. When the bottom clamping unit is engaged and remains as the clamp driving unit while the top clamping unit remains and releases the driving unit, the actuator works in scanning mode and functions as a conventional direct-drive piezoelectric actuator, achieving high-resolution motion. In stepping mode, a large stroke is achieved through cumulative motion steps. Based on this design, the actuator realizes both long-range travel and high resolution via mode switching. A static model of the piezoelectric stack, clamping unit, and driving unit is developed to analyze the output displacement and key structural parameters. Friction between the driving and clamping units is evaluated, and a dynamic model is established for the driving unit. Following this, a prototype actuator is fabricated, assembled, and tested to verify its feasibility and performance. The experimental results confirm that the actuator can achieve self-clamping and self-releasing without an external preload mechanism, enabling bidirectional motion. The maximum driving speed reaches 367 μm/s with a resolution of 0.02 μm, confirming the feasibility of the proposed design.

## Figures and Tables

**Table 1:** PZT stacks parameters.

Parameter	Values
Type	P-887.91
Size of piezo stack	7 mm × 7 mm × 36 mm
Operating voltage	0–120 V
Maximum output	1850 N
Maximum displacement	38 ± 20% μm
Stiffness	50 N/μm
Resonant frequency	40 kHz
Piezoelectric constant	0.108 μm/V
Type	P-888.31
Size of piezo stack	10 mm × 10 mm × 13.5 mm
Operating voltage	0–120 V
Maximum output	3500 N
Maximum displacement	13 ± 20% μm
Stiffness	267 N/μm
Resonant frequency	90 kHz

**Table 2:** Comparison between the proposed actuator and other works.

Reference	Speed (μm/s)	Thrust Force (N)	Step Resolution (μm)	Guiding Structure?
Yang et al. [25]	180	7.6	5.50	No
Deng et al. [18]	155.5	12.3	0.37/0.39	No
Wang et al. [26]	216.3	1.2	N/A	Yes
Ma et al. [19]	471.01	5.88	N/A	Yes
Shao et al. [23]	43	546	0.08	No
This study	367	22	0.02	No

## Data Availability

The original contributions presented in this study are included in the article. Further inquiries can be directed to the corresponding author.
